# Challenges and Long-Term Outcomes of Cementless Total Hip Arthroplasty in Patients Under 30: A 24-Year Follow-Up Study with a Minimum 8-Year Follow-Up, Focused on Developmental Dysplasia of the Hip

**DOI:** 10.3390/jcm13216591

**Published:** 2024-11-02

**Authors:** Marek Drobniewski, Bartosz Gonera, Łukasz Olewnik, Adam Borowski, Kacper Ruzik, George Triantafyllou, Andrzej Borowski

**Affiliations:** 1 Clinic of Orthopaedic and Paediatric Orthopaedics, Medical University of Lodz, 90-419 Łódź, Poland; marek.drobniewski@umed.lodz.pl (M.D.); andrzej.borowski@umed.lodz.pl (A.B.); 2 Department of Clinical Anatomy, Masovian Academy in Płock, 09-402 Płock, Poland; lukaszolewnik@gmail.com (Ł.O.); kacper.ruzik@umed.lodz.pl (K.R.); 3 Medical University of Warsaw, 02-091 Warsaw, Poland; adamborowskio15@gmail.com; 4 Department of Anatomical Dissection and Donation, Medical University of Lodz, 90-419 Łódź, Poland; 5 Department of Anatomy, School of Medicine, National and Kapodistrian University of Athens, Goudi, 157 72 Athens, Greece; georgerose406@gmail.com

**Keywords:** hip arthroplasty, developmental dysplasia of the hip, young population, osteoarthritis

## Abstract

**Background:** Total hip arthroplasty (THA) is a well-established and effective treatment for advanced osteoarthritis (OA) of the hip joint. While commonly performed in older patients, THA is increasingly used in younger individuals, especially in cases of secondary coxarthrosis. Technological advances have led to the development of specialized implants, which allow surgeons to address severe post-inflammatory or dysplastic deformities. Younger patients undergoing THA, often in their 20s or 30s, present higher functional expectations. Despite limited long-term studies, research indicates a higher rate of revision surgeries in this age group compared to older populations, making these procedures a unique challenge. **Methods:** This retrospective study analyzed 5263 primary total hip arthroplasties (THAs) performed at our center between May 1985 and December 2016. After excluding cemented and hybrid implants, as well as patients lost to follow-up or with other etiologies, 101 uncemented THA procedures in 92 patients aged 30 years or younger were included. The majority (64.4%) were due to dysplastic coxarthrosis (DDH), while avascular necrosis (26.7%) and juvenile rheumatoid arthritis (8.9%) accounted for the rest. The average patient age was 25.6 years, with a mean follow-up period of over 24 years. Surgical technique involved the anterolateral approach, with implants placed in the true acetabular region. Implants included Munich/Plasmacup, Mittelmeier, and P-M designs. Implant survival was estimated using the Kaplan–Meier estimator to determine the probability of implant longevity over the follow-up period. Outcomes were assessed using Merle d’Aubigné and Postel scores, modified by Charnley, alongside radiographic evaluations based on the Crowe, De Lee, and Gruen classifications. **Results:** Preoperatively radiological assessment of all hips was classified as grade IV according to the Kellgren–Lawrence scale. Over an average follow-up of 24 years, final outcomes using the modified Merle d’Aubigné and Postel (MAP) classification were excellent in 24%, good in 37%, satisfactory in 8%, and poor in 32% of cases. Results compared between DDH group and control group indicate significantly more poor results for the DDH group compared to the control group (*p*-value < 0.05). All poor outcomes were associated with prosthesis loosening, primarily involving P-M and Mittelmeier acetabular components. Complications included intraoperative fractures in five cases, peripheral nerve dysfunction in six cases, and heterotopic ossification in eight cases. Postoperative pain scores on the VAS scale improved from 6.8 to 1.7. The Kaplan–Meier estimator indicated 10-year survival rates of 85.2% for the entire prosthesis, with 69.8% survival at 15 years and 54.5% at 20 years. For each period the bio-functionality according to Kaplan–Meier estimator was in favor of the control group. **Conclusions:** Cementless THA in patients aged 30 or younger has demonstrated itself to be an efficacious treatment for hip osteoarthritis, yielding favorable bio-functional outcomes in both short- and long-term follow-up. Nevertheless, THA performed in the context of developmental dysplasia of the hip (DDH) carries a significantly elevated risk of postoperative complications, most notably aseptic loosening, which critically undermines implant survival rates. Given the young demographic and the anticipated prolonged functional lifespan of the prosthesis, there is an increased propensity for loosening over time, necessitating vigilant and sustained postoperative surveillance.

## 1. Introduction

Total hip arthroplasty (THA) is currently a widely utilized method and one of the most successful treatments for advanced stages of osteoarthritis (OA) of the hip joint. With the development of endoprosthesis technology, the indications for effectively using this surgical method are increasing. Although endoprostheses are mostly used for elderly patients, this treatment is increasingly utilized in some difficult cases of secondary coxarthrosis and progressively in younger patients [1]. Even severe forms of post-inflammatory or dysplastic degenerative changes in the hip joint can be successfully treated surgically with the skilled application of this method. Multidirectional research by a wide group of specialists on implants has led to the creation of special implants that can be used and can perform their function even in the most advanced deformities of the hip joint [2,3]. Artificial acetabular components with a small diameter, below 44 mm, anatomical stems with a cross-sectional diameter of less than 10 mm, and the modularity of implants allow for the performance of arthroplasty in conditions significantly deviating from the normal anatomy of the human hip joint. It is also noteworthy that the vast majority of patients with secondary coxarthrosis are very young, with significant symptom severity often occurring in the second or third decade of life. The expectations of this group of patients are exceptionally high, given the treatment of osteoarthritis of the hip joint [4]. There are only a few long-term studies regarding arthroplasty in patients under 30 years old, but researchers agree that the percentage of revision surgeries is way higher compared to the older population [4,5,6,7,8]. Therefore, both the complicated intraoperative conditions and the high expectations of patients make arthroplasty procedures in very young patients a unique challenge faced by orthopedic surgeons specializing in THA.

### Purpose

The aim of the study was to analyze the outcomes of total uncemented hip arthroplasty in patients who underwent the surgical procedure before the age of 30, with a follow-up period of at least 8 years and mean follow-up 24 years. Additionally, we wanted to compare the outcomes of procedures performed due to secondary degenerative changes from developmental dysplasia of the hip (DDH) with the outcomes of other arthroplasties conducted before the age of 30 for other reasons.

## 2. Materials and Methods

**Figure 1:**
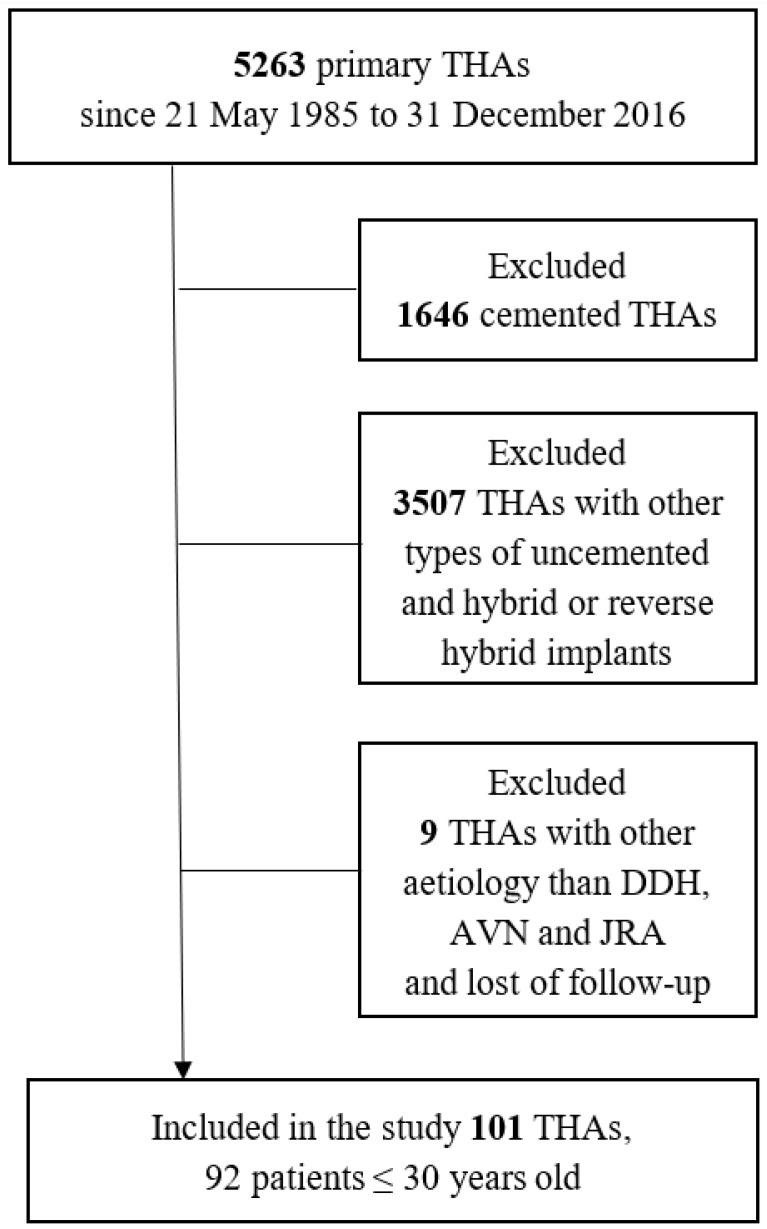
Flow diagram of the distribution of the patient population in the study. THA—total hip arthroplasty; DDH—developmental dysplasia of the hip; AVN—avascular necrosis of the femoral head; JRA—juvenile rheumatoid arthritis.

In our center, total uncemented hip arthroplasty has been performed since May 1985. In the last year, over 600 such procedures were performed. This retrospective study included 5263 primary total hip replacement surgeries performed between 21 May 1985 and 31 December 2016. We have excluded 5153 cemented, hybrid or reverse hybrid implants used for THAs. Then 9 THAs were excluded due to lost follow-up or other etiology than DDH, avascular necrosis (AVN) and juvenile rheumatoid arthritis (JRA). A total of 92 patients aged 30 years and younger, who underwent surgery between 1985 and 2016 due to advanced degenerative changes of the hip joint, with a total of 101 procedures of THA, were included in the study (Figure 1). Of those, 57 women and 35 men were treated. In 55 cases, the left hip joint was operated on, while in 46 cases, the right hip joint was operated on. The average age of all patients on the day of surgery was 27 years (IQR = 13). The youngest patient on the day of surgery was 17 years old, and the oldest was 30 years old. The average follow-up period was 9442 days (IQR = 11,105), which is over 24 years. The average Body Mass Index (BMI) was 26.04 (SD = 4.3).

All procedures were performed under epidural anesthesia, using an anterolateral approach without greater trochanter osteotomy. The acetabular component of the hip endoprosthesis was implanted in the safe zone according to Lewinnek [9]. Typically, the anteversion of the artificial acetabulum did not exceed 15°, and the stem was implanted with a slight ante-torsion of 5–10°. In most cases, the artificial acetabulum was placed in the region of the anatomical location of the primary acetabulum—the True Acetabular Region. The metal cup of the acetabular component was supplemented with a symmetrical (P-M) or asymmetrical 10° (Munich/Plasmacup) acetabular insert made of polyethylene, or the acetabular component was entirely made of ceramic (Mittelmeier). In young patients, operated on before the age of 30, the standard in our center is to use ceramic heads with a diameter of 32 mm.

Standard antibacterial (according to the current epidemiological guidelines) and anticoagulant prophylaxis was implemented in the perioperative period. The day after the procedure, rehabilitative exercises were recommended. After removing the Redon drain, patients began mobilization and learning to walk with simulated or full weight-bearing on the limb, depending on pain tolerance. In the following days, additional rehabilitative exercises were introduced.

The indication for surgical treatment in the study group was secondary advanced osteoarthritis of the hip joint. In 65 cases (64.4%), it was dysplastic coxarthrosis. Additionally, secondary coxarthrosis due to AVN was an indication for hip arthroplasty in 27 cases (26.7%), and degenerative changes due to JRA in 9 cases (8.9%). There were no cases of idiopathic degenerative changes in the study group.

The analysis of the types of implants used showed that the most frequently implanted endoprostheses were Munich or Plasmacup or Screwcup (in 57 cases), Mittelmeier (in 27 cases), and P-M (in 17 cases), with 74 cases using polyethylene–ceramic articulation and 27 cases using ceramic–ceramic articulation. It has to be noted that, due to the conditions imposed by the public healthcare system, we did not have access to all prosthesis available on the market. The detailed characteristics of the study group concerning selected parameters by gender are presented in Table 1.

After hospital discharge, patients were followed up at three-, six-, and twelve-months post-surgery and, subsequently, follow-up visits were conducted once a year. The obtained clinical outcomes were evaluated each time by the same surgeon on every follow-up visit using the classification developed by Merle d’Aubigné and Postel, modified by Charnley (MAP) [10]. This method involves a point-based assessment of pain, gait, and the total range of passive movements within the operated hip joint on a scale of 1 to 6 for each item. In this scale 1 indicates the worst outcome, and 6 suggests best possible outcome. The total maximum score is 18, and the minimum is 3. Pain assessment was performed using a ten-point Visual Analogue Scale (VAS) [11]. In this scale 10-point horizontal line was presented where “0 points” represented “no pain” and “10 points” represented “worst possible pain”. This was a self-assessed scale where patients marked a point on the line that corresponded to their perceived pain level. The probability of implant survival was determined using the Kaplan–Meier estimator [12]. This was used to assess the probability of implant survival at 10, 15, and 20 years. Patients were followed from the date of primary total hip replacement and censored at death or outcome, whichever came first. Outcome was defined as removal or exchange of at least one of the components, including liner exchanges of uncemented cups, for any reason.

Preoperative radiographs were assessed using the Kellgren–Lawrence classification [13] for osteoarthritis and Crowe’s classification for dysplastic coxarthrosis [14]. Radiological examination was also an integral part of follow-up studies. In each case, anteroposterior and axial X-rays of the operated hip joint were taken. The positioning of the endoprosthesis, both the artificial acetabulum and the stem, the degree of implant integration into the bone tissue, and the presence and size of heterotopic ossification were evaluated [15,16]. Additionally, horizontal, vertical, and angular migrations of the acetabular component were assessed. The De Lee and Charnley three-stage classification [17] was used to evaluate the integration of the acetabular component. The Gruen and Moreland classification was used to evaluate the integration of the endoprosthesis stem. The axial positioning of the stem in the proximal femoral metaphysis, signs of vertical migration, bone resorption, hypertrophy, bone density, and the presence of intraosseous and periosteal ossification in seven zones were also assessed [18,19,20]. It is noteworthy that, in all cases, the radiological evaluation was performed by an independent researcher who was not involved in the analyzed surgical procedure.

### Statistical Analysis

Statistical analysis was performed with IBM SPSS Statistics for MacOS, Version 29 (IBM Corp., Armonk, New York, NY, USA). Nominal data between unpaired observations were compared using the Chi-square test, while McNemar’s test was applied for paired observations. Normality was assessed with the Shapiro–Wilk test. Continuous variables were analysed based on measurement type: unpaired measurements were evaluated with an independent *t*-test if normality was met; otherwise, the Mann–Whitney U test was used. A paired *t*-test was employed for paired measurements when normality was satisfied. A *p*-value less than 0.05 was considered statistically significant. The data are presented as Mean (SD) when normality was satisfied; otherwise, they are presented as Median (IQR).

## 3. Results

In the preoperative evaluation, both clinical and radiological results were predictably poor in all cases. All hip joints were classified as grade IV according to the Kellgren–Lawrence classification [13]. On average, over 24 years postoperatively, using the modified MAP classification [10], the following final outcomes were observed: excellent in 24 cases (23.8%), good in 37 cases (36.6%), satisfactory in 8 cases (7.9%), and poor in 32 cases (31.7%)—results compared between DDH group and control group indicate significant differences between the amount of excellent and poor results for each group. The detailed results for each group are summarized in Table 2. The average improvement in final clinical assessment using the modified MAP classification was 6.7 points—a statistically significant improvement. All poor results were associated with prosthesis loosening. Loosening affected both components in 10 cases, the acetabular component alone in 17 cases, and the femoral stem alone in 2 cases. Further detailed analysis indicated that loosening predominantly involved P-M and Mittelemeier acetabular components. Among the poor outcomes, one case involved septic loosening. This patient underwent further surgery to remove the prosthesis and implant an antibiotic spacer, followed by revision arthroplasty two months later, resulting in a good clinical outcome.

There were also a few other complications. Intraoperative fractures of the Adams arch region occurred in five cases, treated with wire cerclage. Peripheral nerve dysfunction complications were observed in six cases: four involving peroneal nerve paresis and two involving femoral nerve paresis. No deaths or thromboembolic complications were recorded. The full list of complications is presented in Table 3.

Radiological assessment, aside from the previously discussed loosening and revision cases, revealed no signs of aseptic prosthesis loosening, with proper femoral component positioning noted. In seven cases (6.9%), undersized femoral stems were implanted, with five cases showing varus positioning and two valgus positioning, attributed to the learning curve. All acetabular components were placed within the original acetabular bone and met Lewinnek’s [9] safe zone parameters. Additionally, heterotopic ossification was noted in eight cases (7.9%), graded as Brooker I in six cases and Brooker II in two cases [15].

Using the VAS scale [11], the average preoperative score was 6.8 points, improving to 1.7 points post-hip arthroplasty, which was a statistically significant improvement. Patients’ subjective assessments post-surgery were significantly better than final outcomes using the modified MAP classification. The greatest improvements were seen in significant pain reduction or elimination and increased range of motion in the operated joint, contributing to overall satisfaction with the surgery. No thigh pain, sometimes observed after cementless hip arthroplasty, was reported. As predicted, relatively worse outcomes were noted in patients treated for coxarthrosis secondary to developmental dysplasia of the hip. However, it is important to note that an excellent rating according to the modified MAP classification reflects a result comparable to that for a healthy hip joint.

Based on the results using the Kaplan–Meier estimator, the probability of implant survival was determined. The 10-year bio-functional survival rate was calculated for 87 cases: 85.2% for the entire prosthesis, 93.8% for the femoral stem, and 86.4% for the acetabular component. The 15-year Kaplan–Meier rate was calculated for 73 cases: 69.8% for the entire prosthesis, 85.7% for the femoral stem, and 71.4% for the acetabular component. The 20-year rate was calculated for 56 cases: 54.5% for the entire prosthesis, 81.8% for the femoral stem, and 56.4% for the acetabular component. The survival rates considering the etiology of hip osteoarthritis are presented in Table 4.

## 4. Discussion

Selecting the appropriate treatment pathway for young and active patients with advanced hip osteoarthritis is considered an extremely challenging task for orthopedic surgeons. Treatment for this patient group must be conducted with exceptional care and should be performed by an experienced surgeon, as the requirements for this age group differ significantly from those of elderly patients. Additionally, hip arthroplasty in individuals under 30 years of age has not been as thoroughly studied as arthroplasty in the elderly. The studies conducted so far have largely been based on small sample sizes and short follow-up periods.

In patients under 30 years of age, advanced hip osteoarthritis is typically secondary in origin and is estimated to affect approximately 1% of the population. Primary—idiopathic—osteoarthritic changes are not observed in these patients. The most common causes of coxarthrosis in young patients include childhood hip disorders, such as developmental dysplasia of the hip, Perthes disease, slipped capital femoral epiphysis, as well as specific and non-specific inflammatory processes. Other causes may include previous hip joint injuries and their complications. Regardless of the origin of the osteoarthritic changes, symptoms related to joint degeneration are often difficult for young patients to accept, and high expectations are placed on the proposed treatment and its final outcome. As these patients are at the peak of their biological, professional, and social activity, a rapid return to full functionality in all aspects of daily life is expected. Currently, the treatment of choice for advanced coxarthrosis in young patients is surgical intervention—total hip arthroplasty (THA). If bone quality allows, the procedure should preferably be performed using a cementless technique with ceramic articulation. The primary goals of hip arthroplasty are to restore a full, pain-free range of motion in the operated joint, relieve pain, and enable a return to a high level of physical activity.

Multiple studies have examined the outcomes of hip arthroplasty in young patients. Adelani et al. [1] reviewed 736 cases, with juvenile rheumatoid arthritis (36.3%) and avascular necrosis (22.6%) the most common indications. Cementless techniques were used in 51.3% of cases. The revision rate was 15.6%, with aseptic loosening the primary cause. Similarly, Walker et al. [21] reported a 5% revision rate in 743 procedures with a functional improvement of 42.17 points over 8.4 years.

Lee et al. [22], in a study of 86 ceramic-on-ceramic hip replacements, found no aseptic loosening after a mean follow-up of 70.8 months. Agrawal et al. [8], analyzing 118 procedures with a mean follow-up of 12.6 years, observed a much higher revision rate of 25%, primarily due to aseptic loosening. However, in Lee et al. [22], the cause of the THA was unknown, while in the Agrawal et al. [8] study it was mostly OA secondary to DDH. Similarly, Mohaddes et al. [7] found that the 15-year bio-functional survival rate was 78%, with younger patients having significantly worse outcomes compared to older ones (78% vs. 89%).

Swarup et al. [23], examined 548 THA in patients under 30 years old with a mean follow-up of 14.2 years, and showed an 87% bio-functional survival at 10 years, dropping to 61% at 20 years. Patients over 25 and males had the best outcomes, particularly with ceramic-on-ceramic or ceramic-on-polyethylene implants. Girard et al. [24] examined THA in the same age group and identified an even higher complication rate. Out of 941 THA, 199 required revision surgery at a mean time 4.6 years after primary THA. On the other hand, Clohisy et al. [25] and Kamath et al. [26] reported great results at 4 and 4.2 years after THA, respectively.

Wangen et al. [27] in 2008 observed no femoral component loosening in 49 procedures over 13 years, though 24 revisions were necessary due to acetabular component failure. Restrepo et al. [28], also in 2008, in 35 cementless procedures, noted only one revision (after 10 years from primary surgery) with average follow up over 6.6 years and substantial pain relief. This strongly suggest that mid-term outcomes are very promising, but bio-functional survival at 10 years decreases significantly.

Finally, Dudkiewicz et al. [29], who have operated only on patients with OA secondary to DDH, found significant functional improvement in all 11 patients (56.9 to 90.6 points) but reported four revisions due to acetabular component loosening, which stands for a 36,4% revision rate. Pruszczyński et al. [30], in 30 procedures, identified seven cases of loosening of the acetabulum in the 6.9 years of follow-up, with better functional outcomes in the remaining patients, although dysplastic coxarthrosis, which was the main cause of the THA (14 cases out 30), led to more complications.

Such results obtained by the previously mentioned authors strongly suggest that OA in the juvenile population is challenging and very often requires revision surgery, especially in cases with longer follow-up (Table 5). Across studies, aseptic loosening of the acetabular component was the leading cause of revisions. It is recommended to utilize ceramic-on-ceramic articulation and a cementless technique, as this is proven to cause fewer complications.

In our study we have distinguished and compared two groups of juvenile patients based on the cause of the THA (1st group includes only DDH as the reason, 2nd group includes other causes). According to our study, OA secondary to DDH is exceptionally challenging among the young patients who require THA.

DDH results in significant alterations in pelvic anatomy, which are critical considerations in hip arthroplasty. High-riding and retroversion of the acetabulum, leg length discrepancy, and femoral neck deformities, such as excessive femoral anteversion, increased neck-shaft angle, and a shortened femoral neck complicate the surgical procedure and necessitate meticulous preoperative planning [31].

It is not only the altered pelvic anatomy that poses a challenge for the surgeon planning hip arthroplasty in a patient with DDH. These patients have often undergone osteotomies in adolescence in order to reposition the pre-arthritic acetabulum or femoral head and delay the onset of secondary osteoarthritis [32]. However, such surgeries result in excessive periacetabular sclerosis, scarring at the surgical access site, and disrupted vascularization in the operated area.

Even with proper surgical technique, the choice of implant is a critical determinant of long-term outcomes in hip arthroplasty. Implant design has a direct impact on the survivorship of an uncemented total hip replacement according to multiple studies. Different cups and femoral stem designs have different contact areas between the implant and bone. The contact area can affect the primary osseointegration process and, even further, the rate of aseptic loosening. In our study, poor outcomes were predominantly associated with the use of the P-M cup, which demonstrated a relatively short bio-functional lifespan due to a tendency for early aseptic loosening, high rates of wear, osteolysis and dislocation [33,34]. This underscores the need for careful selection of implants with proven bio-functional longevity to minimize the risk of revision surgery.

Historically, DDH has been one of the most frequent causes of early hip osteoarthritis, leading to hip arthroplasty at a relatively young age. However, with advancements in diagnostic techniques, particularly the widespread use of neonatal ultrasound screening [35], the incidence of osteoarthritis secondary to untreated DDH has markedly decreased. Moreover, ultrasound screening seems to prevent many other operations and surgical interventions for developmental hip dysplasia [36]. Therefore, some complications during THA due to previous surgeries in the hip region may be avoided.

Given the complexity of the anatomic deformities associated with DDH, standard hip implants may not provide optimal outcomes for all patients. Advances in 3D printing technology offer a novel approach for reconstructing acetabular bone defects that occurred secondary to DDH. Using preoperative 3D CT scans of the patient’s acetabulum, a personalized 3D-printed prosthesis can be created to precisely match the shape of the bone defect. This ensures a highly accurate reconstruction of the defect. Additionally, with the help of specialized software, the internal screw placement for the prosthesis can be meticulously planned, enhancing the safety and reliability of the bone reconstruction process. In the future, the use of custom-made prostheses tailored to the specific anatomy of the individual may become increasingly common [37,38].

So far, there have not been enough studies to definitively demonstrate the superiority of custom-made implants over standard ones in the treatment of coxarthrosis secondary to DDH. Based on the study by Pakos et al. [39], a 10-year implant survival rate of 95.4% can be observed, which is an extremely promising result, but a longer follow-up is needed. Zhang et al. [40] also highlighted several advantages of using custom-made acetabular cups. The primary benefits include restoring bone defects and re-establishing the hip rotation center. However, the authors also emphasize the need for longer-term observation.

### Limitations

The present study does have some limitations, one being that no sample size calculation was performed; however, this study examined the THA in a juvenile population, which is an unusual procedure. Another limitation is that the population was restricted to a Caucasian population from Central Europe, and therefore care should be taken when working with other populations. Moreover, this study was performed retrospectively, therefore an improvement in functional scores could not be ascertained due to a lack of preoperative functional scores.

## 5. Conclusions

Based on the analysis of the obtained results, the following conclusions were drawn: cementless THA is an effective treatment for hip osteoarthritis in patients aged 30 years or younger, providing good bio-functional outcomes in both short- and long-term follow-up. However, THA performed due to DDH compared to JRA and AVN carries a significantly higher risk of aseptic loosening of the prosthesis, substantially affecting implant survival rates. Additionally, due to the young age of the patients and the expected long-term functionality of the implant, there is an increased likelihood of loosening over time, necessitating regular follow-up evaluations over a prolonged observation period.

## Figures and Tables

**Table 1:** Comparison of patients who had THR due to DDH and AVN, JRA. Differences between sex, body sides, age, BMI and mean follow up are examined. *p*-value < 0.05 are considered significant.

	DDH Group 65 Hips, 60 Patients	Control Group 36 Hips, 32 Patients	*p*-Value
Etiology:	DDH—65 hips	AVN—27 hips JRA—9 hips	-
Gender: Female/Male	46/14	11/21	**<0.001**
Side: Right/Left	32/33 hips	14/22 hips	0.224
Age:			0.295
Median (IQR)	27 (13)	26 (13)	
Min.	17	17	
Max.	30	30	
BMI:			**0.024**
Mean	26.04 (SD = 4.3)	23.22 (SD = 3.84)	
Min	18.93	16.94	
Max.	33.22	30.03	
Follow-up (days/years):			**0.025**
Median (IQR)	9442 (11,105)	7650 (11,397)	
Min.	2261 d/6.2 y	1976 d/5.4 y	
Max.	13,366 d/36.6 y	13,373 d/36.6 y	
Implants:			-
Bicontact	31	26	
Mittelmeier	21	6	
P-M	13	4	
Articulation:	21	6	-
ceramic—ceramic			
polyethylene—ceramic	44	30	

DDH—developmental dysplasia of the hip. AVN—avascular necrosis of the femoral head. JRA—juvenile rheumatoid arthritis.

**Table 2:** Final outcomes according to Merle d’Aubigné and Postel, modified by Charnley classification, compared between DDH group and control group (AVN, JRA). *p*-value < 0.05 are considered significant.

MAP Score	DDH Group N (%)	Control Group N (%)	*p*-Value	Total (101 THA)
excellent	7 (10.8%)	17 (47.2%)	**<0.001**	24 (23.8%)
good	25 (38.5%)	12 (33.3%)	0.608	37 (36.6%)
satisfactory	4 (6.2%)	4 (11.1%)	0.377	8 (7.9%)
poor	29 (44.6%)	3 (8.3%)	**<0.001**	32 (31.7%)

MAP score—Merle d’Aubigné and Postel score; THA—total hip arthroplasty; DDH group—patients with developmental dysplasia of the hip; Control group—patients with avascular necrosis of the femoral head or juvenile rheumatoid arthritis.

**Table 3:** Detailed list of complications.

Complication	DDH Group N (%)	Control Group N (%)	*p*-Value
Peroneal or femoral nerve palsy (improved after 3–6 month)	5 (5%)	1 (1%)	0.317
Dislocation	3 (3%)	0	0.751
Superficial wound infection	3 (3%)	1 (1%)	0.650
Deep wound infection	1 (1%)	0	0.668
Intraoperative fracture	5 (5%)	0	0.317
Heterotropic ossifications	8 (7.9%)	0	0.107
Death	0	0	-
Pulmonary embolism	0	0	-
Myocardial infraction	0	0	-
Stroke	0	0	-
Urinary tract infection	0	0	-
Revision surgery for implant malposition	0	0	-
Deep vein thrombosis	0	0	-

DDH group—patients with developmental dysplasia of the hip; Control group—patients with avascular necrosis of the femoral head or juvenile rheumatoid arthritis.

**Table 4:** The Kaplan–Meier bio-functionality coefficient for implants after 10, 15, and 20 years of observation (95% confidence interval), considering the division into the studied subgroups.

	Kaplan–Meyer 10 Years Follow-Up (87 Hips)	Kaplan–Meyer 15 Years Follow-Up (73 Hips)	Kaplan–Meyer 20 Years Follow-Up (56 Hips)
Both elements	85.2%	69.8%	54.5%
	(99.4–76.8)	(83.4–56.3)	(72.4–36.7)
	DDH group (53 hips):	DDH group (44 hips):	DDH group (41 hips):
	81.1%	61.4%	43.9%
	(92.8–69.4)	(79.7–43.0)	(66.8–21.0)
	Control group (28 hips):	Control group (19 hips):	Control group (14 hips):
	92.9%	89.5%	85.7%
	(102.8–83.0)	(104.1–74.9)	(105.5–65.9)
cup	86.4%	71.4%	56.4%
	(94.5–78.4)	(84.6–58.2)	(73.8–38.9)
	DDH group (53 hips):	DDH group (44 hips):	DDH group (41 hips):
	83.0%	63.6%	46.3%
	(94.1–71.9)	(81.5–45.8)	(68.8–23.9)
	Control group (28 hips):	Control group (19 hips):	Control group (14 hips):
	96.4%	94.7%	92.9%
	(103.4–89.4)	(105.1–84.4)	(106.9–78.9)
stem	93.8%	85.7%	81.8%
	(99.2–88.4)	(95.1–76.4)	(93.1–70.6)
	DDH group (53 hips):	DDH group (44 hips):	DDH group (41 hips):
	94.3%	81.8%	80.5%
	(100.7–87.9)	(94.4–69.2)	(94.0–67.0)
	Control group (28 hips):	Control group (19 hips):	Control group (14 hips):
	96.4%	94.7%	92.9%
	(103.4–89.4)	(105.1–84.4)	(106.9–78.9)

DDH group–patients with developmental dysplasia of the hip; Control group–patients with avascular necrosis of the femoral head or juvenile rheumatoid arthritis.

**Table 5:** A comparison of hip arthroplasty outcomes in patients under the age of 30, as reported by various authors between 2002 and 2021.

Author	Year	THA Examined	Cause of the THA	Mean Follow-Up Time	Percentage of Revisions
Adelani et al. [1]	2013	736	JRA, AVN, DDH, trauma	9.5 years	15.6%
Walker et al. [21]	2016	743	AVN, DDH, JRA, Posttraumatic OA, Childhood Hip Sepsis, Legg-Calve-Perthes, SCFE	8.4 years	5%
Lee et al. [22]	2021	86	Not known	5.9 years	0%
Agrawal et al. [8]	2021	118	DDH, JRA	12.6 years	25%
Mohaddes et al. [7]	2019	504	DDH, AVN, JRA, Arthritis, Primary OA	15 years	
Swarup et al. [23]	2018	548	AVN, DDH, JRA, Posttraumatic OA, Childhood Hip Sepsis, Legg-Calve-Perthes, SCFE, others	14.2 years	
Clohisy et al. [25]	2010	102	AVN, secondary arthritis	4.2 years	6.9%
Kamath et al. [26]	2012	21	AVN, DDH	4 years	4.8%
Wangen et al. [27]	2008	49	DDH, AVN	13 years	49.0%
Restrepo et al. [28]	2008	35	AVN, JVR, DDH	6.6 years	2.9%
Dudkiewicz et al. [29]	2002	11	DDH	9 years	36.4%
Pruszczyński et al. [30]	2011	30	DDH, AVN, JRA	6.9 years	23.3%

THA—total hip arthroplasty; JRA—juvenile rheumatoid arthritis; AVN—avascular necrosis of the femoral head; DDH—developmental dysplasia of the hip; OA—osteoarthritis; SCFE—slipped capital femoral epiphysis.

## Data Availability

The raw data supporting the conclusions of this article will be made available by the authors on request.

## Abbreviations

THA—total hip arthroplasty; OA—osteoarthritis; DDH—developmental dysplasia of the hip; JRA—juvenile rheumatoid arthritis; AVN—avascular necrosis; SD—standard deviation, BMI—The Body Mass Index, MAP—Merle d’Aubigné and Postel, modified by Charnley.
